# Mr. Rickards, on Cancerous Affections

**Published:** 1801-12

**Authors:** J. Rickards

**Affiliations:** Member of the Royal College of Surgeons, London. New Brentford


### *To the Editors of the Medical and Phyfical Journal.*

GENTLEMEN,

IN the laft Number of your very valuable Mifcellany, I obferve the detail of a Cafe of Cancer of the Cheek, in the perfon of Mr. Knight of Friendsbury, related by Mr. Atkinfon of Iflington; wherein the firft appearances and infidious progrefs of that truly dreadful difeafe, is defcribed with fufficient accuracy to give any one who has had much opportunity of obfervation in chirurgical practice, as good an idea of the nature of the difeafe as could be gained by ocular examination. I fhould not prefume to offer any obfervations on this cafe, were it not that Mr. Atkinfon feems to exprefs a wifh that fome of your Correfpondents would take it into confideration, and "probably fuggeft fome means for alleviating the fufferings" of the patient.

It would afford me great fatisfaction, if the obfervations which I have to offer had a "probability" of alleviating fuch fufferings as a perfon labouring under fo fevere and terrible a difeafe muft endure. By the account given by your Correfpondent, this cafe feems not to have been underftood in the firft inftance; the primordium of the difeafe appears to have been fmall, and of little moment; an infulated fpeck of morbid action, or of morbid ftructure, the *complete and entire* removal of which by the knife, an operation attended with no difficulty if timely performed, might have removed the difeafe altogether; for I believe it is now generally agreed by Surgeons, that Cancer, in its origin, is a difeafe entirely local, not proceeding from, or dependent on, any acting power or morbid affection fituated in the fyftem or general conftitution; that its morbid actions are, in the firft inftance, confined to its own limits; that it has the power, after a time, by a fpecific inflammation, of generating a fpecific poifon, which, by being received, by means of the abforbents that arife from the difeafed part, into the fyftem, has the power of affecting the parts through which it paffes, with the fame kind of difeafe as that from which itfelf was produced.

While, therefore, a difeafe of this kind remains in an occult, indolent ftate, in a ftate of fcirrhus merely, not producing matter which may be abforbed; while it is perfectly circumfcribed, not extending any degree of induration or thickening to the furrounding parts; and when, from its peculiar fituation, it is within the reach of the knife of the Surgeon, there can be no queftion as to the mode of conduct to be recommended. We cannot always fucceed in our endeavours to perfuade patients for their benefit in fuch cafes; for while they experience little or no trouble from a difeafe, they are frequently unwilling to fubmit to an operation for its removal. But were the nature of the cafe fairly ftated to them, and the difmal picture of what they have every reafon to expect, placed before their view, there are, I believe, few individuals who would refufe to embrace fafety from fuch a terrible malady, at the expence of a few moments tranfitory pain. After all, if a patient remains obftinate, no blame can be attached to the practitioner; but if fuch operation is not propofed at fuch time only as it may avail any thing, he is certainly very open to cenfure.

In the prefent ftate of this difeafe, removal by the knife is quite impracticable, and certainly not to be thought of; indeed, it is extremely difficult to fay, what line of conduct we fhould purfue in fuch a fevere affliction. When the difeafe has advanced to fuch a dreadful ftate, it is generally judged ufelefs even to have recourfe to any remedy adapted to cure the difeafe, but merely to direct our practice to fuch means as will, from time to time, afford fome degree of eafe and comfort to the fufferer.

I fhould not have troubled you, Gentlemen, and the public, with thefe obfervations, but from the very eminent advantage which I have feen refult from one remedy in cafes of ulcerated Cancer, particularly, I think, when fituated in the face. The remedy is by no means new; but it is not, I believe, (where I have had an opportunity of obferving the practice of others) fo frequently ufed as its merits demand. The remedy I allude to is the Arfenic, as a cauftic. Among other cafes in which I

have have witnessed decided advantage from its application, I will take the liberty briefly to mention one which was under my care about two years since.

The master of a fishing-smack had for several months a considerable induration of the under lip, which by degrees became painful, and continued to increase in size and hardness for some time; at length the pain became excessive, ulceration began, and spread rapidly; he then, but not before, applied for advice. When I first saw him, the greater part of the under lip (extending from the left angle of the mouth to beyond the centre of the lip) was in a state of ulceration, exhibiting the true characteristic appearances of Cancer, the whole surface being filled with those peculiar fungous excrescences which arise from cancerous tumours, frequently bleeding at the slightest touch; the boundaries, for a considerable extent, of a stony hardness, and much inflamed; the pain excruciating and unintermitting; the ability to chew his food nearly lost from the stiffness of his jaw; the saliva running continually down his chin. In this state I determined to make a trial of the arsenic, but feared, from the surrounding hardness, that the disease was too extensive to admit of removal. I mixed some of a powder, composed of arsenic, sulphur, &c. (nearly similar to the celebrated formula of Plunkett) with a little white of an egg, and smeared it over the whole of the external part of the lip, as far as the Cancer extended, avoiding as much as possible the inner part of the lip; for which purpose I insinuated a piece of lint between the teeth and lip, and advised him not to swallow any of his saliva. The pain at first was considerable, but soon abated; a deep eschar was soon formed, which in a few days began to separate. When that process was complete, it left a sore, discharging thick matter, instead of the thin, cancerous virus which before exuded from it; the indurations were likewise much lessened. But thinking the caustic had not penetrated sufficiently deep, I repeated its application a second, and again a third time. By the period of the separation of the third eschar, the whole lip, nearly, (except a portion toward the right angle), was destroyed by the caustic; the sore, however, exhibited a clean and healthy appearance; all the induration in the cheeks and chin, which before felt like stone, was entirely vanished; all pain was at an end; the cavity soon filled with healthy granulations, and his lip very speedily was healed, and scarcely shewed a trace or scar of the disease which had existed.

This disease was certainly not nearly so extensive as that of Mr. Knight, and it may be thought that they bear no comparison; but, surely, in a disease of so terrible a nature, any hope is better than resigning a patient to his fate. In the case just

 related, related, I had little *hopes* of fuccefs; but had defpair gained afcendancy over that fmall ray of hope, this patient, in all probability, would have lingered out a painful exiftence under a loathfome malady.

From what I have feen of arfenical applications in this and various other cafes, it appears to me that they poffefs a fpecific power in the deftruction of the cancerous fungus; certain it is that the efchar made by it in cancerous ulcers is much deeper than that which it will produce in any other kind of ulceration. Suppofe, in the prefent cafe, (if it fhould be thought worth a trial) this cauftic were to be applied to a fmall portion only of the tumour at firft, to mark its effects: If this efchar fhould feparate kindly, and the pain fhould be fufferable, the application might be repeated again and again, more or lefs freely, according to its effects.

With refpect to the internal adminiftration of this mineral in cafes of open cancerous affections, I can fay but little from my own experience; I have given it in thefe cafes, but it has never been continued a fufficient length of time, or taken with fufficient accuracy, to warrant a conclufion for or againft its utility: But we are authorized, from the obfervations of a correfpondent of great accuracy and refpectability in a late number of your Journal, to conclude that the powers of Arfenic, internally exhibited, are very efficacious in thefe difeafes, of which he has given us fome well-marked Cafes.

I beg leave again to obferve, that I fhould not have taken the liberty to offer an opinion on any cafe under the care of other gentlemen, but from Mr. Atkinfon's wifh in his letter.

I am, your's, &c.

*New Brentford, Nov. 9, 1801.*

J. RICKARDS, Member of the Royal College of Surgeons, London.

